# Platycodin-D exerts its anti-cancer effect by promoting c-Myc protein ubiquitination and degradation in gastric cancer

**DOI:** 10.3389/fphar.2023.1138658

**Published:** 2023-03-06

**Authors:** Qianqian Xu, Guangzhao Pan, Zhonglan Wang, Lingling Wang, Yancheng Tang, Jinyun Dong, Jiang-Jiang Qin

**Affiliations:** ^1^ School of Pharmaceutical Sciences, Zhejiang Chinese Medical University, Hangzhou, China; ^2^ Zhejiang Cancer Hospital, Institute of Basic Medicine and Cancer (IBMC), Chinese Academy of Sciences, Hangzhou, Zhejiang, China; ^3^ School of Life Sciences, Tianjin University, Tianjin, China; ^4^ Key Laboratory of Prevention, Diagnosis and Therapy of Upper Gastrointestinal Cancer of Zhejiang Province, Hangzhou, China; ^5^ School of Chinese Medicine, Hong Kong Baptist University, Hong Kong SAR, China

**Keywords:** Platycodin D, c-Myc, ubiquitination, cell apoptosis, gastric cancer

## Abstract

Platycodin D (PD) is a triterpene saponin extracted from the root of *Platycodon grandiflorum.* It has been reported to exhibit multiple pharmacological and biological properties. There is substantial evidence to support that PD displays a wide range of anti-tumor activities. However, the detailed molecular mechanism still needs further elaboration. In the present study, to explore whether PD inhibits gastric cancer (GC) cell viability, eight GC cell lines and the GES-1 cell line (a gastric mucosal cell line) were tested. We found that PD exhibited better inhibitory activity on GC cell lines than on the non-tumor cell line. Besides, treatment with PD led to a significant cell cycle arrest, thereby causing subsequent apoptosis. Regarding the cell growth inhibition mechanism, PD can downregulate the protein level of c-Myc rather than its mRNA level in a dose-dependent manner. Further studies revealed that PD disturbed the overall ubiquitination level in GC cell lines and enhanced the ubiquitination-dependent degradation of c-Myc. Interestingly, the inhibition of cell viability by PD could be restored to a certain extent when the expression of c-Myc was recovered, suggesting that PD-mediated GC cell growth inhibition is closely associated with c-Myc expression. Our study proposes a novel molecular mechanism for PD inhibiting GC cell proliferation and growth by destabilizing the c-Myc protein. This work may lay a preliminary foundation for developing PD as an anti-cancer therapy.

## Introduction

Gastric cancer is the fifth most commonly diagnosed cancer and the third leading cause of cancer-related deaths worldwide (Sung et al., 2021; Ajani et al., 2022; Hu et al., 2022). Despite recent advances in the management of GC, including surgery combined with radiotherapy and chemotherapy, the prognosis for advanced GC patients remains poor, and postoperative treatments such as medication-assisted treatment are vital for patients. However, the lack of effectiveness, low toxicity, and specific anti-GC agents are still urgent problem that needs to be solved.

The cell cycle is a highly regulated process that can promote cell growth, genetic material replication, and division (Suski, Braun, Strmiska, &Sicinski, 2021). In normal cells, the cell cycle is an accurate regulatory process, strictly controlled by cell-cycle-dependent transcription and protein degradation and several CDK inhibitor proteins (Evan and Vousden, 2001). Unlike normal cells, the cell cycle of tumor cells is highly specialized, making it possible for tumor cells to proliferate almost indefinitely (Evan & Vousden, 2001). Therefore, targeting cell-cycle proteins seems to be a potential strategy to combat tumor growth. Despite the current success with some cell cycle inhibitors, such as CDK4/6i (Palbociclib, Ribociclib, and Abemaciclib) (Fry et al., 2004; Finn et al., 2009), CDK2i (Milciclib) (Otto & Sicinski, 2017), CDC7i (Iwai et al., 2019), these cell-cycle targeted cancer therapeutic strategies are still in its infancy (Alvarez-Fernandez & Malumbres, 2020).

As a transcription factor, the c-Myc protein is an early discovered oncogene with cell transformation function (Garcia-Gutierrez et al., 2019). The Myc family comprises three proteins: c-Myc, L-Myc, and N-Myc. It is noteworthy that Myc is highly expressed through different mechanisms in 60%–70% of human solid and hematopoietic tumors; also, the high or abnormal expression of Myc is closely associated with poor prognosis and the degree of deterioration (Duffy, O'Grady, Tang, & Crown, 2021; Llombart & Mansour, 2022; Moon, Park, & Ro, 2021). Growing evidence has indicated that the major oncogenic mechanism of c-Myc is the promotion of cell cycle progression and the disturbs of cell cycle regulation (Freeman-Cook et al., 2021; Hsin, Shen, Chang, Ko, & Wang, 2021; Liu et al., 2021; Yao et al., 2022). Myc regulates the cell cycle in multiple ways; for one thing, it promotes or starts the process of the cell cycle by activating or inducing cyclins (including D-type cyclins, E-type cyclins, cyclin A and B1), CDKs (including CDK1, CDK2, CDK4, and CDK6), and E2F transcription factors (including E2F1, E2F2, and E2F3). Another thing it inhibits a set of proteins called cell-cycle brakes to ensure the continuous progress of cell division (Garcia-Gutierrez et al., 2019). Therefore, due to the typical cancer-driven feature, Myc has been regarded as an ideal drug target (C. Wang et al., 2021). Undoubtedly, the long-term pursuit of Myc inhibitors has always been the long-cherished wish of researchers. However, directly targeting the Myc oncoprotein is yet hopeful and challenging. Three major problems need to be considered. First, as a universal transcription factor, Myc is widely expressed in normal and tumor tissues and unexpected inhibition of Myc normal cells may cause side effects. Secondly, the structure of Myc protein has no optimal binding pocket and binding sites for traditional small-molecule inhibitors. Finally, the nuclear localization of the Myc protein makes it hard to develop antibody drugs for this target (Pelengaris, Khan, & Evan, 2002; Wang et al., 2021). Hence, alternative approaches need to be further explored to circumvent these obstacles.

**FIGURE 1:**
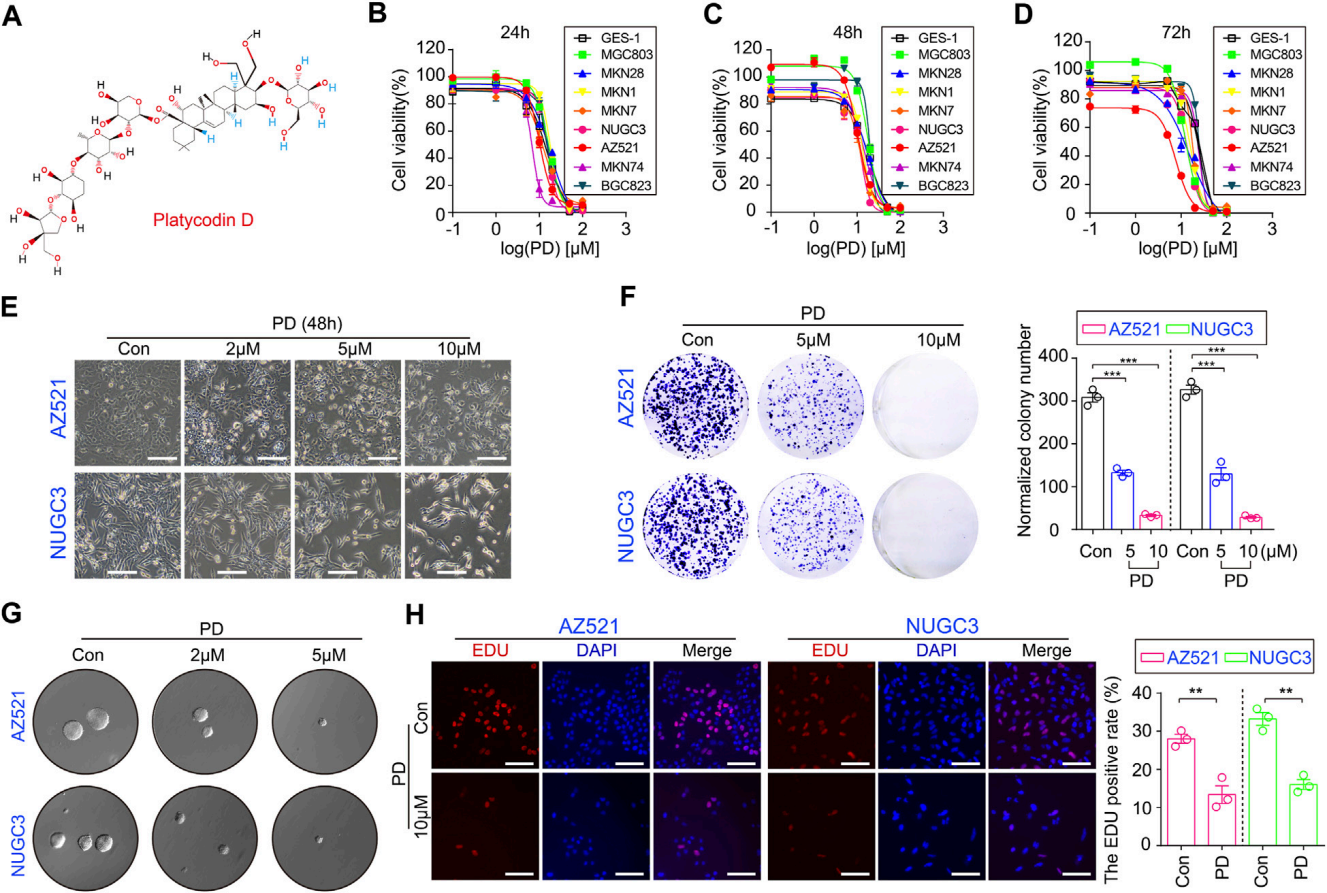
PD inhibits cell growth and colony formation in gastric cancer cell lines. **(A)** The schematic diagram shows the chemical structure of PD. The CCK-8 assays were performed to assess the IC_50_ values of PD after treating cells for 24 h **(B)**, 48 h **(C)**, and 72 h **(D)**. **(E)** The diagram shows the morphologies of AZ521 and NUGC3 cells treated with PD at different concentrations (0, 2, 5, or 10 μM) for 48 h **(F)** The clone numbers of each group were shown by a histogram. **(G)** The soft agar assay was performed to examine the proliferation and colony formation ability of AZ521 and NUGC3 cells after treatment with DMSO (control group) or the indicated concentrations of PD. **(H)** The images of EDU staining in AZ521 and NUGC3 cells treated with DMSO (control group) or 10 μM PD for 48 h. Scale bar = 100 μm. The EDU positive rates of each group were shown by a histogram. All data are mean ± SEM and are representative of three independent experiments. *p < 0.05, **p < 0.01; ***p < 0.001.

We aimed to make efforts to screen the superior Myc inhibitors for gastric cancer therapy. As the “cradle” of drug screening, drug developers increasingly favor natural products due to their excellent therapeutic and low side effects. Here, Platycodin-D (PD) **(** Figure 1A **)**, isolated from the dry root of *Platycodon grandiflorum,* exhibits a strong anti-cancer effect. Recent studies have shown that PD has anti-inflammatory (Wang, Guo, &Wang, 2019), anti-cancer, anti-aging (Shi, Li, &Zhang, 2020), anti-virus, and other medicinal properties (Khan, Maryam, Zhang, Mehmood, &Ma, 2016). However, the tumor suppressive activity of PD and its mechanisms of action in gastric cancer has not been well elaborated. In this study, we found that PD exhibits significant anti-cancer activity *in vitro*. Treatment with PD resulted in substantial cell cycle arrest and apoptosis. Mechanically, PD upregulated the whole ubiquitination levels in gastric cancer cells. Further studies revealed that PD could promote the degradation of oncoprotein c-Myc through the ubiquitin-proteasome system (UPS). Our study proposes a molecular mechanism that PD inhibits gastric cancer cell proliferation and growth by destabilizing c-Myc. This work not only elucidates the molecular mechanisms for PD’s anti-cancer effects but also lays a preliminary foundation for anti-cancer drug discovery and development.

## Results

### PD inhibits the growth and colony formation ability in gastric cancer cell lines

To examine PD’s anti-GC activity *in vitro*, we assessed its effects on GC cell viability using CCK-8 assay. We observed that PD treatment at different concentrations for 24 h markedly reduced the viability of multiple gastric cancer cell lines (Figure 1B), while the normal gastric mucosal cell line (GES-1) appeared to be more resistant to PD, as shown by its IC_50_ values in different cell lines (Supplementary Figure S1A). We next treated gastric cancer cell lines and GES-1 cells with the same concentrations of PD for 48 h (Figure 1C) and 72 h (Figure 1D), respectively. The CCK-8 analyses showed that PD could reduce cell viability in both a concentration- and time-dependent manner (Figures 1B–D, Supplementary Figures S1B, C). The morphological changes in responses to the treatment of PD in both AZ521 and NUGC3 cell lines have also been shown (Figure 1E, Supplementary Figures S1D). Moreover, the plate colony and soft agar colony formation assays showed that PD significantly inhibited gastric cancer cell proliferation in a dose-dependent manner (Figures 1F,G). Using the EDU staining, we observed that PD caused a significant reduction of EDU-positive cells in AZ521 and NUGC3 cell lines (Figure 1H). Altogether, these results suggested that PD can inhibit gastric cancer cell growth and colony formation *in vitro*.

### PD mediates cell division disorder and causes cell cycle arrest

**FIGURE 2:**
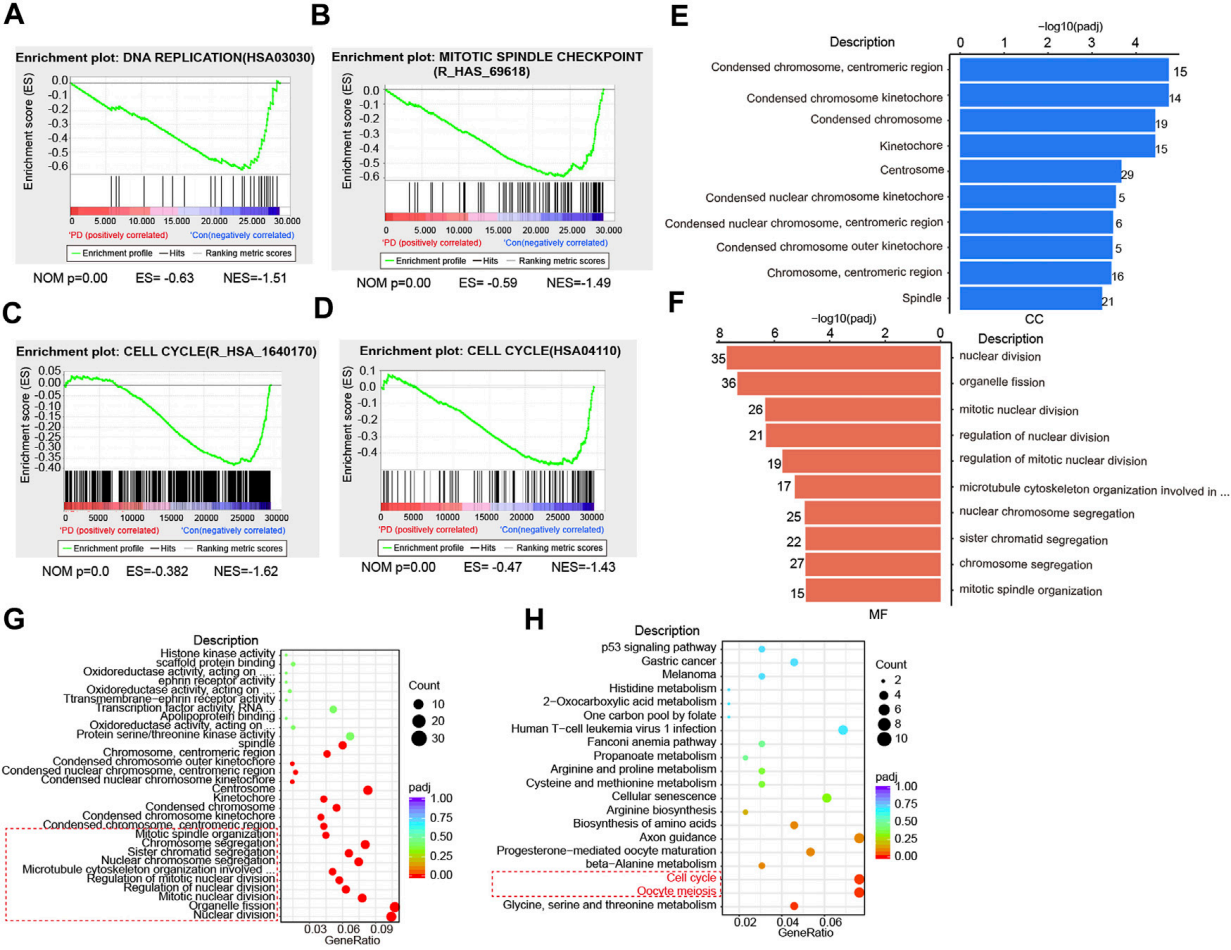
PD treatment affects cell division and cell cycle progression based on RNA-seq analysis. **(A)** GSEA analysis of the DNA replication in PD-treated (10 μM) and DMSO-treated cells based on RNA-seq results. **(B)** GSEA analysis of the mitotic spindle checkpoint in PD- (10 μM) and DMSO-treated cells. **(C, D)** GSEA analysis of the cell cycle (R_HSA_1640170 and HSA04110) in PD- (10 μM) and DMSO-treated cells. GO analysis of cellular components (CC) **(E)**, molecular functions (MF) **(F)**, and biological processes (BP) **(G, H)** in PD- (10 μM) and DMSO-treated cells.

To explore the underlying mechanisms for PD’s anti-cancer activity, we next performed RNA-sequencing (RNA-seq). The gene set enrichment analysis (GSEA) showed that PD treatment induced a significant DNA replication disorder (Figure 2A). Further analysis based on the data set of mitotic spindle checkpoints indicated that the PD-treated GC cells exhibited pronounced downregulation of the related pathways compared with the control group (Figure 2B). Moreover, the enrichment analysis of datasets R_HSA_1640170 and HSA04110 indicated that PD-treated cells showed significant downregulation of cell cycle-related genes (Figures 2C,D). Therefore, we inferred that the potential mechanisms for the inhibition of GC cell proliferation by PD might be related to cell cycle arrest.

It has been reported that cell division disorder may be associated with changes in cell structure and properties (Huang et al., 2022), such as the structural destruction of the chromosome, the extent of destruction of the nucleus, cytoskeleton, and other components (Moore et al., 2021; Yilmaz et al., 2021). To investigate whether PD treatment could induce changes in cell components, we analyzed the possible changes in cell composition after PD treatment. We found that the chromosome constituents in gastric cancer cells were changed significantly by PD treatment (Figure 2E), including condensed chromosome, centromeric region, condensed chromosome kinetochore, and spindle et al. We also noticed that those molecular functions (MF) or biological processes (BP), including nuclear division, organelle fission, mitotic nuclear division, chromosome segregation, and mitotic spindle organization, were changed significantly (Figures 2F,G). The Gene Ontology (GO) Enrichment analysis also showed that PD caused cell cycle disorder (Figure 2H). Altogether, these results consistently demonstrated that PD treatment affected the cell cycle process, which might be essential for its anti-cancer activity.

### PD induces G1 phase arrest and apoptosis through the downregulation of the p21/CDK2-CyclinE signaling pathway

**FIGURE 3:**
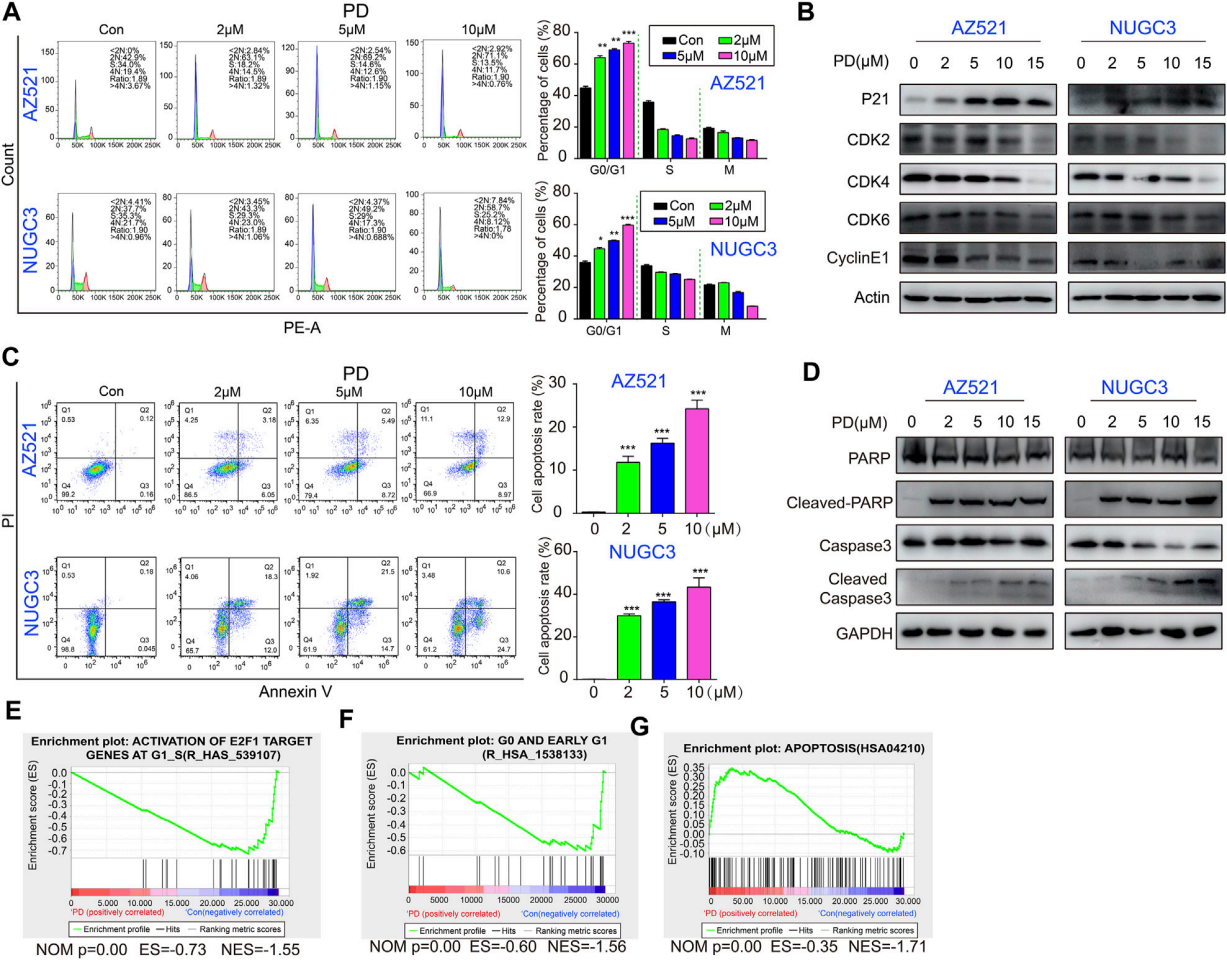
PD induces cell cycle arrest and causes subsequent apoptosis in gastric cancer cells. **(A)** The cell cycle distribution of AZ521 and NUGC3 cell lines was examined by flow cytometry after treatment with PD (0, 2, 5, and 10 μM) for 48 h. The percentages of different phase cells were shown by a histogram. **(B)** Western blotting assays were performed to detect the expression of p21, CDK2, CDK4, CDK6, and CyclinE1 in AZ521 and NUGC3 cell lines after treatment with PD (0, 2, 5, 10, and 15 μM) for 48 h. **(C)** The apoptotic rates of AZ521 and NUGC3 cell lines were evaluated by flow cytometry after treatment with PD (0, 2, 5, and 10 μM) for 48 h. The cell apoptosis rates were shown by a histogram. **(D)** Western blotting assays were performed to detect the expression of PARP, cleaved-PARP, Caspase 3, and cleaved Caspase-3 in AZ521 and NUGC3 cell lines after treatment with PD (0, 2, 5, 10, and 15 μM) for 48 h. **(E)** GSEA analysis of the activation of E2F1 target genes at G1_S in PD- (10 μM) and DMSO-treated cells based on RNA-seq results. **(F)** GSEA analysis of the G0 and early G1 phase in PD- (10 μM) and DMSO-treated cells. **(G)** GSEA analysis of apoptosis in PD- (10 μM) and DMSO-treated cells. All data are mean ± SEM and are representative of three independent experiments. *p < 0.05, **p < 0.01; ***p < 0.001.

We further examined PD’s effects on cell cycle distribution by flow cytometry analysis. The results showed that PD treatment for 48 h effectively induced cell cycle arrest at the G1 phase in gastric cancer cells in a concentration-dependent manner (Figure 3A). Meanwhile, PD decreased the protein levels of CDK2, CDK4, CDK6, and Cyclin E1 and increased the level of p21 in a concentration-dependent manner (Figure 3B). These findings suggested that PD-induced cell cycle arrest at the G1 phase might be due to the modulation of the p21/CDK2-CyclinE pathway.

We next assessed whether PD induces apoptosis using flow cytometry analysis. As expected, PD treatment significantly induced apoptosis in gastric cancer cell lines in a dose-dependent manner (Figure 3C). Besides, apoptosis-related proteins (including cleaved-PARP and cleaved Caspase3) were also markedly increased (Figure 3D). Moreover, the GSEA analysis of the RNA-seq data showed that PD treatment decreased the enrichment of genes related to the G1 phase (Figures 3E,F). On the contrary, the PD treatment group enriched several apoptosis-related genes (Figure 3G). In conclusion, PD induced cell cycle arrest and apoptosis by inhibiting the p21/CDK2-CyclinE signaling pathway.

### PD decreases the protein level of c-myc by activating the ubiquitin degradation pathway

**FIGURE 4:**
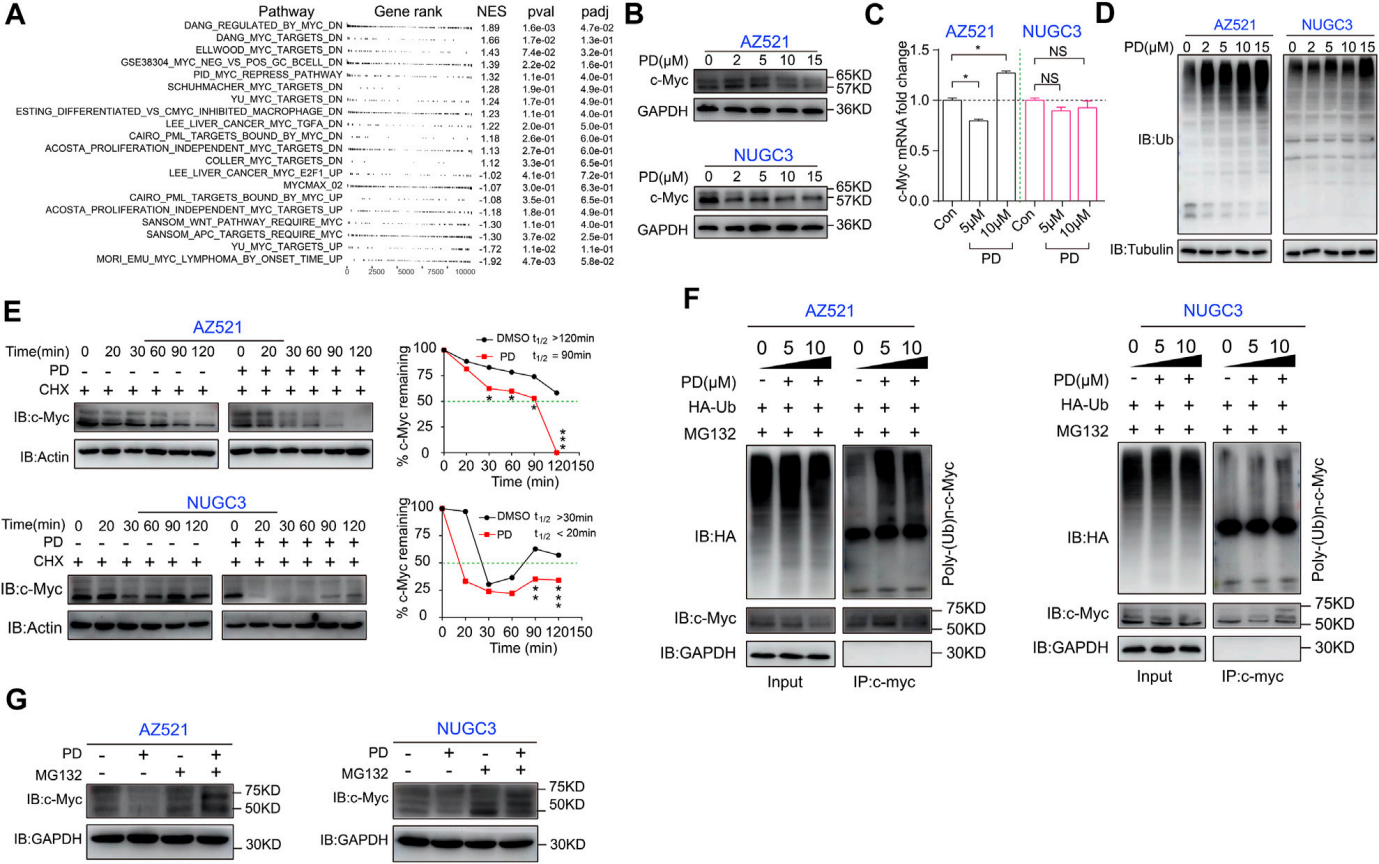
PD induces ubiquitination and proteasomal degradation of c-Myc in gastric cancer cells. **(A)** GSEA analysis of the Myc pathway in PD-(10 μM) and DMSO-treated GC cells based on RNA-seq. UP, upregulation; DN, downregulation. **(B)** Western blotting assays were performed to detect the expression level of c-Myc in AZ521 and NUGC3 cells after treatment with PD (0, 2, 5, 10, and 15 μM) for 48 h. **(C)** qRT-PCR was performed to detect the relative mRNA level of c-Myc in AZ521 and NUGC3 cells after treatment with PD (0, 5, and 10 μM) for 48 h. The DMSO-treated cells were used as the control group. **(D)** Western blotting assays were performed to detect the ubiquitin (Ub) expression level in AZ521 and NUGC3 cells after treatment with PD (0, 2, 5, 10, and 15 μM) for 48 h. **(E)** Western blotting assays were performed to detect the expression level of c-Myc in AZ521 and NUGC3 cells after treatment with CHX (Cycloheximide) or co-treatment with CHX and PD. The relative level of c-Myc was shown by a line chart. **(F)** PD-treated AZ521 and NUGC3 cell lysates were immunoprecipitated with antibodies against c-Myc and then immunoblotted for ubiquitination. **(G)** Western blotting assays were performed to detect c-Myc levels in AZ521 and NUGC3 cells treated with DMSO or PD (10 μM) in the presence or absence of MG132 (10 μM) for 48 h. All data are mean ± SEM and are representative of three independent experiments. *p < 0.05, **p < 0.01; ***p < 0.001.

We next explored the molecular mechanisms responsible for PD’s effects on cell cycle- and apoptosis-related proteins. The GSEA analysis hinted that the oncogene c-Myc-related pathways changed significantly after PD treatment (Figure 4A). Therefore, we detected PD’s effects on c-Myc expression at both the mRNA and protein levels. Western blotting analysis showed that PD treatment decreased the protein level of c-Myc in gastric cancer cell lines in a dose-dependent manner (Figure 4B). At the same time, there was no apparent alteration in its mRNA level (Figure 4C). In this case, we had to consider that PD might affect the post-transcriptional modification of c-Myc. We further found that the overall ubiquitination level in PD-treated cells was increased in a concentration-dependent manner (Figure 4D), suggesting that PD might induce the degradation of c-Myc through ubiquitination. We performed a protein half-life experiment to determine whether PD affects the stability of c-Myc. As shown in Figure 4E, PD treatment caused a shorter half-life of c-Myc in a time-dependent manner in both AZ521 and NUGC3 cell lines.

Generally, when ubiquitination is activated, the substrate can be specifically recognized and labeled by ubiquitin, thereby bounding and degrading by a proteasome named 26S (Aliabadi, Sohrabi, Mostafavi, Pazoki-Toroudi, & Webster, 2021). To validate whether PD could recruit ubiquitin to c-Myc, we next performed an immunoprecipitation (IP) assay. As shown in Figure 4F, PD treatment could recruit the exogenously expressed ubiquitin (HA-tag labeled ubiquitin) in both cell lines in a concentration-dependent manner. Moreover, the protein level of c-Myc could be rescued by MG132 (a proteasome inhibitor that can effectively block the proteolytic activity of the 26S proteasome complex) (Lee, Park, & Nam, 2021) (Figure 4G). Altogether, these results indicated that PD could accelerate the ubiquitination and degradation of c-Myc.

### PD inhibits GC cell growth by blocking the c-myc/p21/CDK2-Cyclin E pathway

**FIGURE 5:**
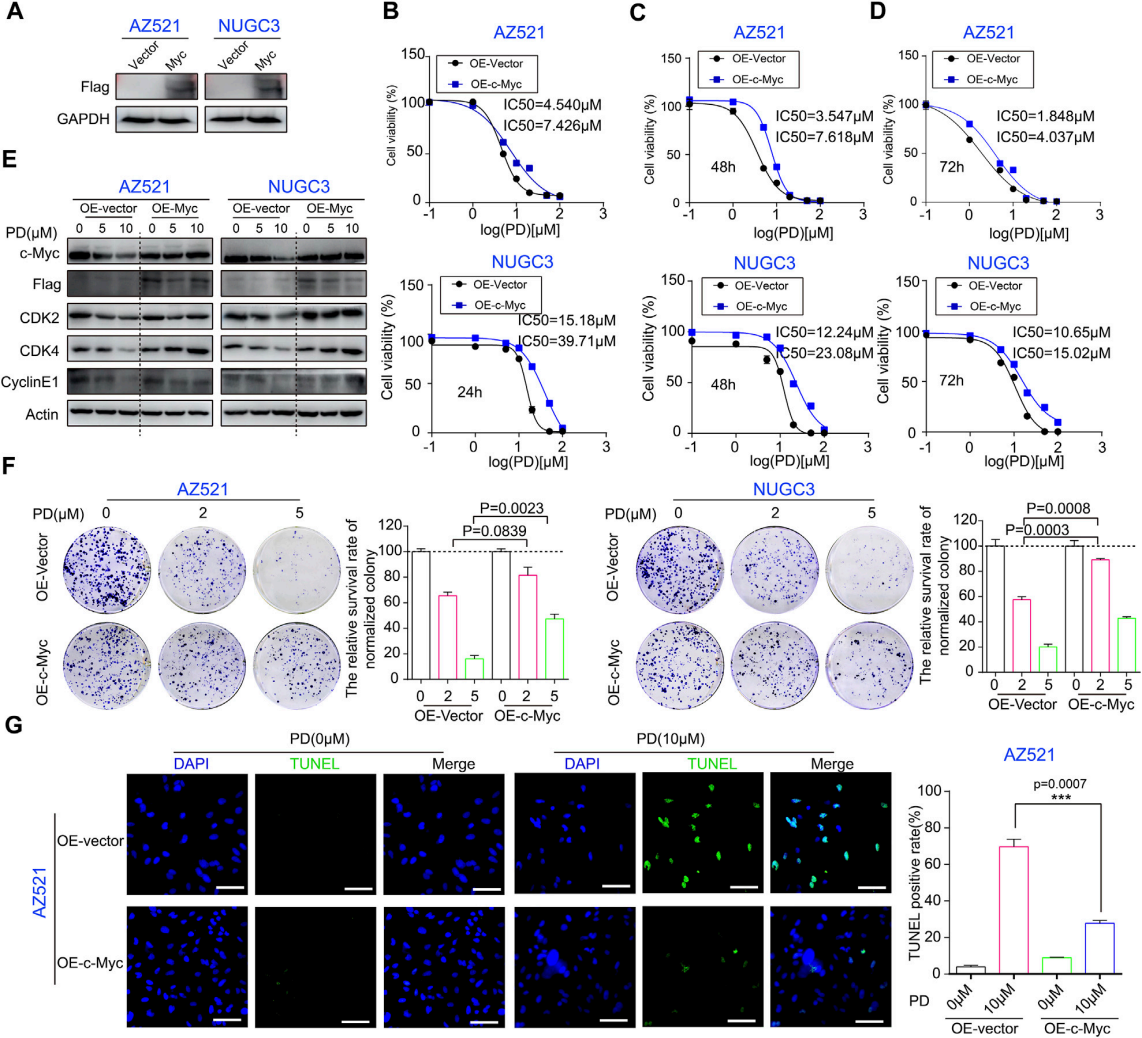
c-Myc expression plays an important role in PD’s anti-cancer activity. **(A)** Western blotting assays were performed to detect the exogenous expression level of c-Myc in AZ521 and NUGC3 cells. The CCK-8 assays were performed to assess the IC_50_ values of PD after treatment with Myc- or vector-overexpressing cells for 24 h **(B)**, 48 h **(C)**, and 72 h **(D)**. **(E)** Western blotting assays were performed to detect the expression levels of c-Myc, CDK2, CDK4, Cyclin E1, and exogenous Myc protein (Flag marked) in AZ521 and NUGC3 cells after overexpressing Myc or vector. **(F)** Plate colony formation assays were performed to examine the proliferative ability of Myc overexpressing or control AZ521 and NUGC3 cell lines after treatment with DMSO (control group) or the indicated concentrations of PD. The relative survival rate of colonies was shown by a column chart. **(G)** The TUNEL staining was performed to assess the cell apoptosis rate in Myc- and vector-overexpressing AZ521 cells after treatment with DMSO or PD (10 μM) (Scale bar = 100 μm). The TUNEL-positive rates of cells were counted by a column chart. All data are mean ± SEM and are representative of three independent experiments. *p < 0.05, **p < 0.01; ***p < 0.001.

We further determined the role of c-Myc in PD’s anti-cancer activity through a series of rescue experiments. A Myc overexpression vector was constructed to rescue the downregulation of c-Myc by PD treatment (Supplementary Figure S2A). The Myc stable overexpression gastric cancer cell lines were successfully developed using the lentivirus-mediated infection system (Figure 5A). Subsequently, the CCK-8 assay was performed to evaluate the IC_50_ values of PD in gastric cancer cell lines overexpressing Myc or vector. Compared with the vector group, the IC_50_ values of PD in Myc-overexpressing cell lines significantly increased, indicating the enhanced resistance of GC cell lines to PD treatment due to Myc overexpression (Figures 5B–D).

Next, we examined the levels of these cell cycle-related proteins in Myc-overexpressing cell lines to determine the role of Myc in PD’s inhibitory effects on the p21/CDK2-Cyclin E pathway with or without PD treatment. As shown in Figure 5E, the expression of G1 phase proteins was downregulated by PD in the vector group. However, Myc overexpression significantly reduced the effects of PD on these proteins. The plate cloning experiments displayed that the Myc-overexpressing cell lines formed larger clones (Figure 5F), and the colonies of the vector group exhibited a lower survival rate (Figure 5F). Moreover, the TUNEL staining results indicated that Myc overexpression significantly reduced PD-mediated apoptosis in AZ521 cells (Figure 5G). In conclusion, these findings demonstrated that the c-Myc signaling pathway is critical in PD’s anti-cancer activity.

## Discussion

Gastric cancer is a major contributor to cancer incidence and mortality worldwide (Yeoh and Tan, 2022). Drug therapy runs through the whole process of gastric cancer treatment. However, the lack of adequate and specific drugs is still a severe problem (Yan et al., 2022). In the present study, we found that Platycodin D (PD) (Figure 1A), isolated from the dry root of *P.latycodon grandiflorum,* exhibits a superior anti-cancer effect. This work may provide a foundation for developing PD as an anti-GC agent.

Abnormal cell cycle progression is a common feature of all tumors and a major driving force for tumorigenesis (Suski et al., 2021). Therefore, targeting the cell cycle is a feasible therapeutic strategy for cancer (Ahmad et al., 2020; Piezzo et al., 2020; Susanti & Tjahjono, 2021). The first-generation CDK inhibitors had been developed and exhibited broad activity upon CDKs. Subsequently, a new generation of particular ATP-competitive CDK4/6 inhibitors (such as ribociclib, palbociclib, and abemaciclib) was developed. These inhibitors can induce reversible G1 phase arrest in retinoblastoma-positive tumor models (Ingham and Schwartz, 2017). CDK4/6 inhibitors seem to be the most promising of the cell cycle therapeutics. However, these inhibitors’ toxicity and subsequent tolerability cannot be ignored (O'Leary et al., 2016). For several decades, the world has witnessed an overwhelming interest in natural compounds for their massive pharmaceutical potential in combating cancer (Varghese & Dalvi, 2021). Natural compounds are called the “cradle” of new drugs because of their low toxicity and high efficacy. Therefore, natural products have displayed broad application prospects in tumor suppression, inflammation inhibition, virus, and bacterial combat, and other functions (Cui and Jia, 2021; Hu et al., 2022; Scotti & Scotti, 2022; Zhang, Li, Si, &Xu, 2021). Previous studies have reported that PD possesses multiple biological and pharmacological properties, including anti-nociceptive (Liu et al., 2020), anti-atherosclerosis, antiviral, anti-inflammatory, anti-obesity, immunoregulatory, hepatoprotective, and anti-tumor activities (Huang et al., 2019; Khan et al., 2016). PD has been reported to suppress tumor growth and metastasis through multiple mechanisms, such as cell cycle arrest, apoptosis induction, and autophagy activation. Chun et al. reported that PD could inhibit AGS cell proliferation and induce cell anoikis through phosphatidylserine externalization, DNA fragmentation, sub-G1 phase arrest, and caspase activation. They further elaborated that p38 activation is the primary mechanism for PD-induced apoptosis in AGS cells (Chun, Joo, Kang, & Kim, 2013). Another report indicated that miR-34a played an essential role in gastric cancer progression, which could enhance the susceptibility of GC to PD by targeting survivin (Peng, Fan, Xiong, Lou, & Zhu, 2019). However, the underlying mechanism of PD against gastric cancer needs further exploration.

In this study, we discovered that PD treatment significantly caused G1 phase arrest and subsequent apoptosis (Figure 3). Our study demonstrated that PD inhibits gastric cancer cell proliferation and growth by destabilizing the c-Myc protein. Myc is famous as an oncoprotein and is highly expressed in multiple cancers (Baluapuri, Wolf, &Eilers, 2020; Yao et al., 2022). The Myc protein is a potential therapeutic target due to the high dependence on Myc for tumor growth maintenance (Baluapuri et al., 2020). Therefore, Myc exhibits excellent research value and has attracted the extensive attention of many researchers. Myc’s most typical function is coordinating transcription elongation with DNA replication and cell cycle progression (Baluapuri et al., 2020). Here, we found that PD could increase the whole ubiquitination level in the cells. Further studies revealed that PD could promote the degradation of c-Myc through UPS (Figure 4). We also found that PD-mediated Myc degradation caused the inactivation of the p21/CDK2-CyclinE pathway. However, the E3 ligase responsible for PD-induced ubiquitination of Myc is unknown and needs further exploration.

**FIGURE 6:**
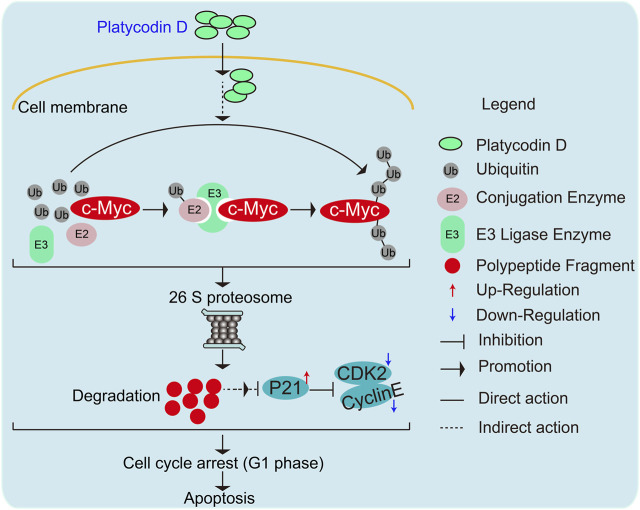
Schematic diagram elucidating the molecular mechanisms responsible for PD-mediated gastric cancer cell growth inhibition and apoptosis.

Of note, we also validated the importance of c-Myc expression for the anti-cancer activity of PD (Figure 5). In future work, we will explore whether PD suppresses tumor growth through the above mechanisms using animal models. In conclusion, our research proposed a novel mechanism that PD regulates c-Myc protein stability through UPS (Figure 6). This work demonstrated a molecular mechanism responsible for the anti-cancer activity of PD. It may provide a basis for developing this natural product as a novel and effective anti-cancer therapeutic agent.

## Materials and methods

### Cell lines and reagents

The gastric cancer cell lines MKN74, BGC-823, MKN1, MKN7, NUGC3, AZ521, MGC-803, and MKN28, and a normal gastric cell line GES-1 were procured from the American Type Culture Collection (ATCC). MKN74, BGC-823, MKN1, MKN7, NUGC3, MGC-803, MKN28, and GES-1 cell lines were maintained in RPMI 1640 (Gibco; Thermo Fisher Scientific, Waltham, MA, United States), and grown at 37 °C with 5% CO_2_. The AZ521 cells were cultured in MEM basic, which contained 10% FBS (Fetal Bovine Serum, Bio-Channel), 1% Penicillin-Streptomycin Solution (Biosharp^®^ life science, China), 1% Sodium Pyruvate (Gibco; Thermo Fisher Scientific, Waltham, MA, United States), and 1% MEM Non-Essential Amino Acids (Gibco; Thermo Fisher Scientific, Waltham, MA, United States). The Myc overexpression stable NUGC3 cell lines were cultured in RPMI 1640, which contained 15 μg/mL puromycin (P9620; Sigma-Aldrich St; Louis, MO, United States). The Myc overexpression stable AZ521 cell lines were incubated in MEM medium containing 10 μg/mL puromycin. Platycodin D was bought from Herbest Biotechnology Co., Ltd. (Xi’an, China). Cycloheximide (HY-12320) and MG132 (HY-13259) were purchased from MedChemExpress (MCE). C-Myc (#9402), GAPDH (#5174), Ub (#3936), p21 (#2947), CDK2 (#18048), CDK4 (#12790), CDK6 (#13331), Cyclin E1 (#20808), PARP (#9532), Caspase 3 (#9662), and cleaved-Caspase 3 (#9664) antibodies, the second antibody anti-mouse IgG, HRP-linked antibody (#7076), anti-rabbit IgG, and HRP-linked antibody (#7074) were acquired from Cell Signaling Technology (CST). Tubulin antibody was a product of Beyotime (AF1216). The HA antibody was purchased from Abcam (ab1424).

### CCK-8 assay

Briefly, the cells (2×10^3^ cells/well) were plated onto the 96-well plates, and the cells were treated with different concentrations of PD for 24 h, 48 h, or 72 h. After treatment, the cells were incubated with 10 μL of CCK-8 solution at 37°C for 3.5 h. The results were acquired by a detection instrument (SPATRK, TECAN) and analyzed using GraphPad Prism 6 (Qi et al., 2022; Yu et al., 2022).

### EdU staining

3×10^4^ cells were plated onto 24-well plates. The next day, cells were treated with 10 μM PD at 37°C for 48 h. The cells treated with the same volume of DMSO were regarded as the control group. The EDU agents (C0078S, BeyoClick™ EdU Cell Proliferation Kit with Alexa Fluor 594; Beyotime) were added and incubated with cells for 2 h. The cells were washed with 1× PBS and fixed in 4% paraformaldehyde for 10 min. The EDU staining was performed according to our previous study (Pan et al., 2022). At least three microscopic fields were required to record the percentages of EDU staining.

### Soft agar colony formation and plate clone formation assays

Soft agar colony and plated clone formation assays were applied to evaluate the effects of PD on colony formation and cell proliferation ability. For soft agar assay, 1.5 mL of the MEM or RPMI 1640 medium containing 0.6% agarose and different concentrations of PD were pre-added in the 6-well plates. The mixture of 2×10^3^ cells and 1.5 mL medium (MEM or RPMI 1640 medium containing 0.3% agarose and different concentrations of PD) was added to each well. After 23 days, 200 μL of methylthiazolyldiphenyl-tetrazolium bromide (MTT) was added into each well and cultivated for 30 min at 37°C. Photographs were recorded using a digital camera.

For plate clone formation assay, 3 mL of MEM or RPMI 1640 medium containing 2×10^3^ cells were seeded in a 6-well plate. After 1 day, cells were co-incubated with different concentrations of PD at 37°C for 13 days. The medium was then removed, and 1× PBS was added to each well for washing. The crystal violet (C0121; Beyotime) solution was used for cell staining for 45 min at room temperature. Photographs were recorded using a digital camera (Yuan et al., 2021; Dong et al., 2022).

### RNA-seq and data analysis

The AZ521 cells were incubated with 10 μM PD or the same volume of DMSO for 48 h. Then the samples were harvested, and total RNA was isolated from cells using a Trizol assay. Transcriptome sequencing and analysis of RNA samples were performed by Novogene company (Beijing, China). The TruSeq stranded mRNA sample preparation kit (Illumina Inc., United States) was adopted to prepare the RNA libraries using 250 ng of RNA. Subsequently, the RNA libraries were sequenced by a HiSeq platform (Illumina, San Diego, CA, United States) on a 150-bp paired-end run. The differentially expressed genes were screened by a *p*-value cut-off for a false discovery rate (FDR) of 0.05 and a minimum 2-fold change in expression, as described previously (Zhang et al., 2018).

### Quantitative real-time PCR

The AZ521 and NUGC3 cells were incubated with PD at 37°C for 48 h. The total RNA was isolated using RNA-Quick Purification Kit (YiShan Biotechnology Co. LTD., Shanghai, China). Fast-All-in-One RT Kit (YiShan Biotechnology Co. LTD., Shanghai, China) was applied to synthesize the first-strand cDNA following the manufacturer’s protocol. The qRT-PCR assay was carried out by a CFX96TM Real-Time System (Bio-Rad Laboratories, Hercules, CA, United States) with the following reaction mixture: 2x Super SYBR Green qPCR Master Mix (10 µL) (YiShan Biotechnology Co. LTD., Shanghai, China), forward and reverse primers (0.5 µL), nuclease-free water (7 µL), and cDNA (2 µL) (Wang et al., 2018; Zhang et al., 2020). All primers were acquired from PrimerBank (https://pga.mgh.harvard.edu/primerbank/) and are shown in Supplementary Table S1. GAPDH was used as an internal control.

### Western blotting and immunoprecipitation (IP) assays

The PD-treated cells were collected, and the total protein was extracted using RIPA buffer (Beyotime, P0013K) containing phosphatase inhibitor (Abcam, ab201112) and phenylmethanesulfonyl fluoride (PMSF, Solarbio^®^ Life Science). BCA assay (Enhanced BCA Protein Assay Kit, Beyotime, P0009) was applied to measure the protein concentrations. As described previously, 8%–12.5% SDS-PAGE gels were performed to separate the protein bands. Protein bands were transferred to polyvinylidene fluoride (PVDF) membranes (Millipore, United States). After that, the PVDF membranes were incubated with 5% skim milk (BioFROX, 1172GR500) at room temperature for 2 h and then co-incubated with primary antibodies at 4°C, followed by incubation with anti-mouse IgG, HRP-linked antibody or anti-rabbit IgG, HRP-linked antibody at room temperature for 2 h. The protein bands were acquired using the detection instrument (AMERSHAM ImageQuant 800, Cytiva (GE)) (Li et al., 2020; Tian et al., 2021).

For the immunoprecipitation (IP) assay, the PD-treated cells were collected and dissolved in 1 mL cell lysis buffer (CST, #9803). The sample was treated with 2.5 μL rabbit IgG and 50 μL protein A + G Agarose (Beyotime, P2055) at 4°C for 4 h and then centrifuged at 3,500 rpm for 5 min. The supernatants were collected, 20 μL c-Myc antibody was added into the supernatant, and the mixtures were incubated at 4°C overnight. The next day, 50 μL protein A + G Agarose was added and incubated at 4°C for 4 h. All samples were centrifuged at 3,500 rpm for 5 min. The precipitates were collected and washed with 1× PBS three times. Finally, 50 μL protein loading buffer was added to the tube and incubated at 100°C for 15 min. The proteins of interest were detected through Western blotting assay as described above.

### Flow cytometry

The cell cycle and apoptosis assays were performed as described previously (Li et al., 2022; Xu et al., 2022). Briefly, the PD-treated cells were harvested, washed, and fixed. After washing away unbound dye, the PI and RNase A (Sigma Aldrich, United States) were added and incubated for 45 min at 37°C. Subsequently, the samples were subjected to flow cytometry LSRFortessa (BD Bioscience, United States), and the results were analyzed using the FlowJo_V10 software (Three Star, Ashland, OR, United States). For the cell apoptosis assay, the cells were resuspended in 1×binding buffer, and then FITC-labeled Annexin V (BD Pharmingen, United States) was added and incubated for 15 min following the manufacturer’s protocols. The percentage of apoptotic cells was detected by the flow cytometer CytoFLEX LX (Beckman Coulter, United States). The results were analyzed using FlowJo_V10 software.

### TUNEL staining

3×10^4^ cells were plated onto 24-well plates. The next day, the cells were incubated with 10 μM PD for 48 h at 37°C. The cells treated with the same volume of DMSO were regarded as the control group. According to the manufacturer’s protocols, the One Step TUNEL Apoptosis Assay Kit (C1086, Beyotime) was applied to perform the TUNEL staining. A fluorescence microscope (Nikon, 80i, Nikon Corporation, Tokyo, Japan) was used to observe and record photographs.

### Statistical analysis

The data are presented as mean ± SEM. Data were analyzed by GraphPad Prism 6.0 software, and the significant differences between groups were detected by the Student’s t-test. The asterisks indicate statistically significant differences (*p < 0.05, **p < 0.01, and ***p < 0.001).

## Data Availability

The datasets presented in this study can be found in online repositories. The names of the repository/repositories and accession number(s) can be found in the article/Supplementary Material.
