# Supplementary material for: Platycodin-D exerts its anti-cancer effect by promoting c-Myc protein ubiquitination and degradation in gastric cancer
Source: Front Pharmacol. 2023 Mar 6;14:1138658. doi: 10.3389/fphar.2023.1138658 (PMC10025306; doi:10.3389/fphar.2023.1138658)
Supplement: Supplementary file 1 [file DataSheet1.doc]

***Supplementary Material***

**Platycodin-D exerts its anti-cancer effect by promoting c-Myc protein ubiquitination and degradation in gastric cancer**

Qianqian Xu^1,2†^, Guangzhao Pan^2,4†*^, Zhonglan Wang^1,2†^, Lingling Wang^2,3^,

Yancheng Tang^5^, Jinyun Dong^2,4^, Jiang-Jiang Qin^1,2,4*^

1. School of Pharmaceutical Sciences, Zhejiang Chinese Medical University, Hangzhou, China
2. Zhejiang Cancer Hospital, Institute of Basic Medicine and Cancer (IBMC), Chinese Academy of Sciences, Hangzhou, Zhejiang 310022, China
3. School of Life Sciences, Tianjin University, Tianjin, China
4. Key Laboratory of Prevention, Diagnosis, and Therapy of Upper Gastrointestinal Cancer of Zhejiang Province, Hangzhou, China
5. School of Chinese Medicine, Hong Kong Baptist University, Hong Kong SAR 999077, China

* Correspondence: Jiang-Jiang Qin, [jqin@ucas.ac.cn](mailto:jqin@ucas.ac.cn)

Guangzhao Pan, guangzhaousing@163.com

† These authors have contributed equally to this work

**Key words:** Platycodin D, c-Myc, ubiquitination, cell apoptosis, gastric cancer

**Running title:** Platycodin D promotes C-Myc degradation

**Supplementary Data**

**Supplementary S1. PD inhibits cell growth and self-renewal ability in GC cell lines. (A)** The IC_50_ of PD treated BGC-823, MKN74, GES-1, MKN1, MKN7, NUGC3, AZ521, MGC803, and MKN28 cells with different concentrations for 24 h. **(B)** The IC_50_ of PD treated BGC-823, MKN74, GES-1, MKN1, MKN7, NUGC3, AZ521, MGC803, and MKN28 cells with different concentrations for 48 h. **(C)** The IC_50_ of PD treated BGC-823, MKN74, GES-1, MKN1, MKN7, NUGC3, AZ521, MGC803, and MKN28 cells with different concentrations for 72 h. **(D)** The heat map demonstrates that the cell viability of AZ521 and NUGC3 cells treated PD at different concentrations (0, 0.1, 1, 5, 10, 20, 50, and 100μM) for 48 h. Plate colony formation assay was performed to investigate the proliferation of AZ521 and NUGC3 cells after treatment with DMSO (control group) or the indicated concentrations of PD.

**
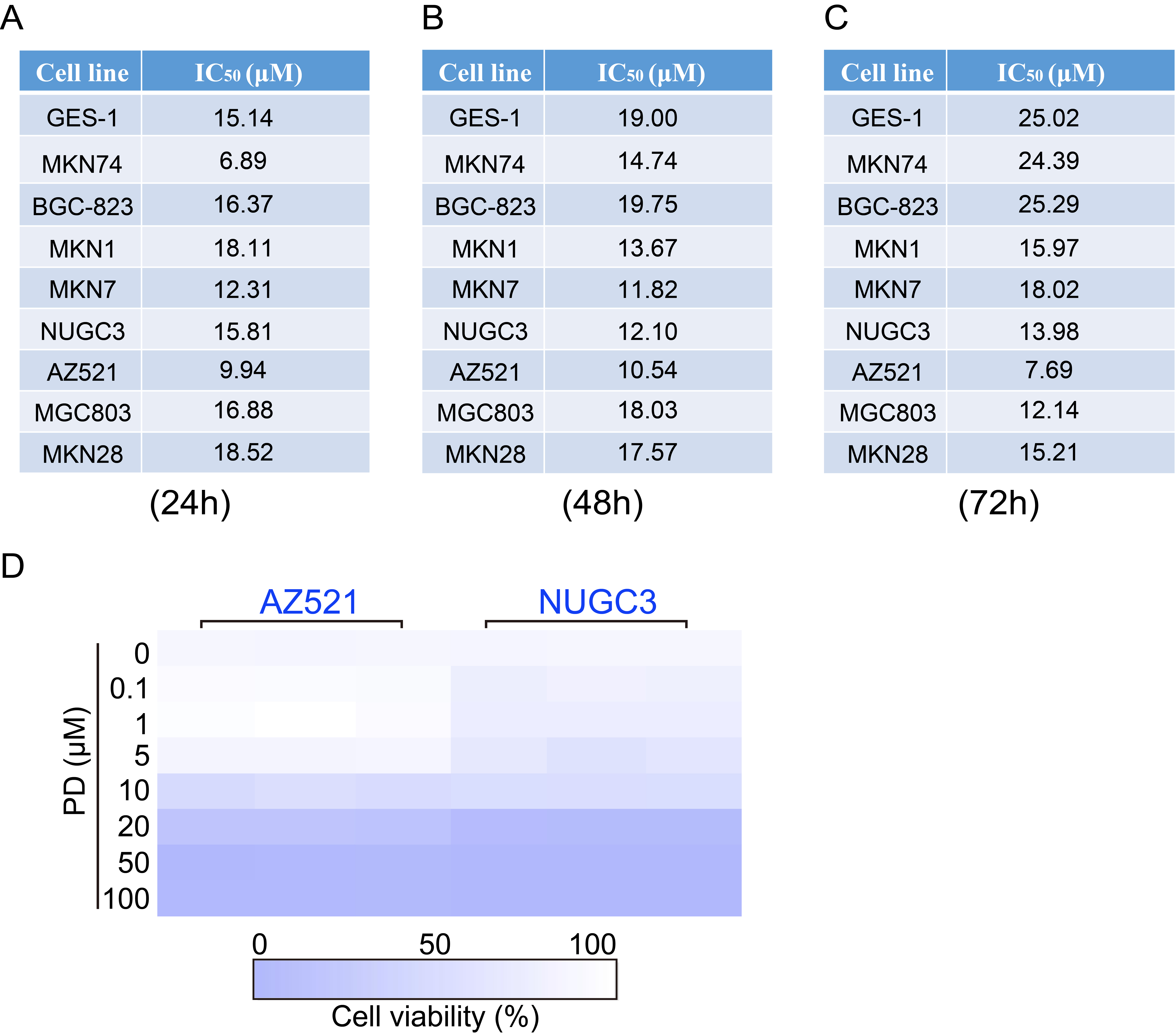
**

**Supplementary S2. The schematic diagram of c-Myc overexpression vector construction.**

**
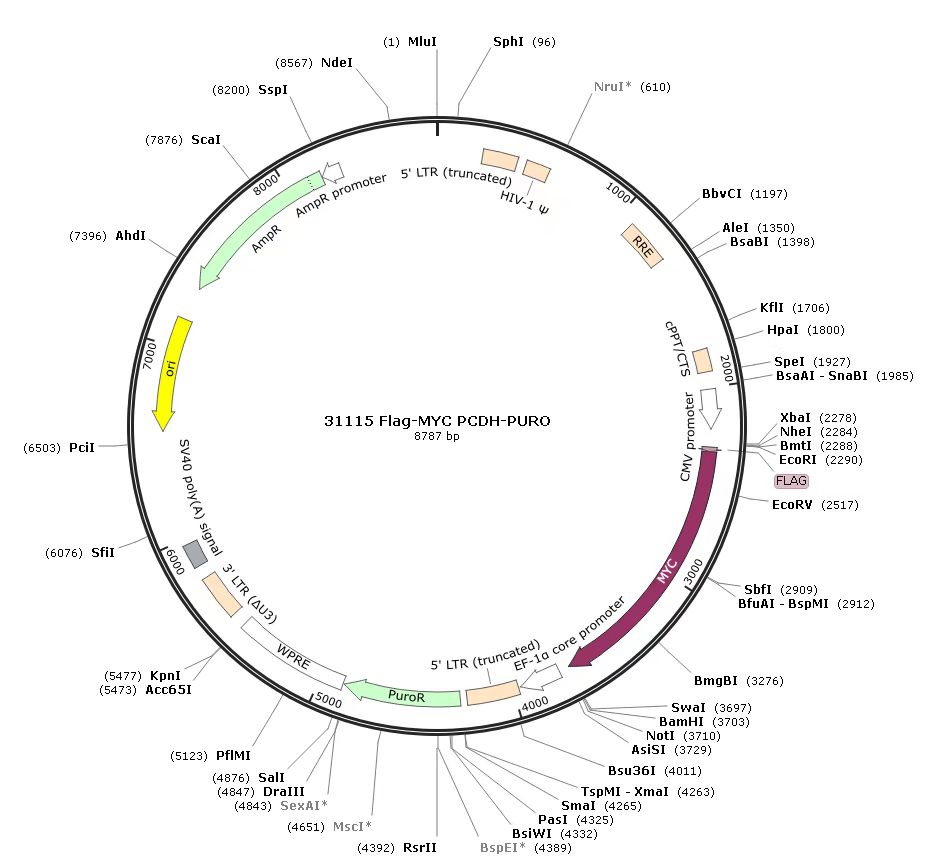
**

**Table S1 The detailed sequences of qRT-PCR used in this study.**

| **Primer name** | **Sequences (5’-3’)** |
| --- | --- |
| c-Myc-qRT-PCR-F | TCCCTCCACTCGGAAGGAC |
| c-Myc-qRT-PCR-R | CTGGTGCATTTTCGGTTGTTG |
| GAPDH-F | ACAACTTTGGTATCGTGGAAGG |
| GAPDH-R | GCCATCACGCCACAGTTTC |

*****The raw data of the RNA-seq can be acquired in *NCBI GEO* database, and the GEO accession code is [*GSE225649*](https://www.ncbi.nlm.nih.gov/geo/query/acc.cgi?acc=GSE225649).

*The raw data of this manuscript is showed in jianguoyun ([https://www.jianguoyun.com/](http://links.email.frontiersin.org/ls/click?upn=AAaFa03elZRFPXQ6ShiKwAPom0YBz5uwBQPZjOWa130qeDbhxfpxOsMI3cHpBqBw7gB9_ud7YfarPDemVXpsifiCgfHCwQWsmhTkbwlQIji3mrs5WRa3K9cagOrCV1URkpe7m-2BPmVFBeo3dVB59m0Y8I-2FCZ-2FSHOe1J5pyP96E1KwEJaBL1MLi-2FHBB2O-2FxC1Lz-2BgffDiGJkn9DpoZueuzHaa2jkVpBwmbv28-2Bdb3PP-2FCGlwjhy84NDYRfTNnRPwiXVl-2BzwomyQqe43bmP2u34GMDXtYnlQYOksOTZwBEiCgsyc6jpNUy-2FVzt-2F65wF7b0QRgL7hpwblacc8fAEnXMIEesKjpE4Lv6CB1pr6ACt1s7o5W2L2h2ENqx31rUC7yoq5q-2FZB)), please click the link ([https://www.jianguoyun.com/p/Df4nk-YQo-OnCxinj_EEIAA) to](https://www.jianguoyun.com/p/Df4nk-YQo-OnCxinj_EEIAA）to) view.
